# Dietary patterns of children aged 6–24 months assisted by the Bolsa Família Program

**DOI:** 10.1017/S1368980021004110

**Published:** 2021-10-01

**Authors:** Marília Moura e Mendes, Giovana de Montemor Marçal, Ana Elisa Madalena Rinaldi, Nassib Bezerra Bueno, Telma Maria de Menezes Toledo Florêncio, Ana Paula Grotti Clemente

**Affiliations:** 1 Nutrition Postgraduate Program, Federal University of São Paulo, Paulista School of Medicine, Botucatu St., 740, Vila Clementino, 04023-062 São Paulo, Brazil; 2 Faculty of Nutrition, Federal University of Alagoas, A.C. Simões Campus, Maceió, AL, Brazil; 3 Faculty of Medicine, Federal University of Uberlândia, Uberlândia, MG, Brazil

**Keywords:** Infant nutrition, Complementary feeding, Social vulnerability, Principal component analysis

## Abstract

**Objective:**

This study aimed to verify the association between socio-economic and demographic characteristics and dietary patterns (DP) of children assisted by the Conditional Cash Transfer Program, Bolsa Família Program (BFP).

**Design:**

This is a cross-sectional study. DP were defined using a principal component analysis. The association of the predictive variables and DP was modelled using multilevel linear regression analysis.

**Setting:**

This study was conducted in six municipalities from the State of Alagoas, Brazil.

**Participants:**

The participants were children aged 6–24 months who were assisted by the BFP.

**Results:**

A total of 1604 children were evaluated. Four DP were identified (DP1, DP2, DP3 and DP4). DP1 is composed of traditional Brazilian food. DP2 is formed mostly from ultra-processed foods (UPF). DP3 consists of milk (non-breast) with added sugar, while DP4 consists of fresh and minimally processed foods. Caregivers with higher age and education (*β* = −0·008; (95 % CI −0·017, −0·000); *β* = −0·037; (95 % CI −0·056, −0·018), respectively) were negatively associated with DP2. We observed a negative association between households with food insecurity (*β* = −0·204; (95 % CI −0·331, −0·078)) and DP4 and a positive association between caregivers with higher age and education (*β* = 0·011; (95 % CI (0·003; 0·019); *β* = 0·043; (95 % CI 0·025, 0·061), respectively) and DP4.

**Conclusion:**

This study identified the association between socio-economic inequities and DP early in life, with an early introduction of UPF, in children assisted by BFP in the State of Alagoas.

Adequate and healthy complementary feeding in the first years of life guarantees child growth, development, and health and thus represents an important aspect of health-related quality of life^(1–3)^. The introduction of healthy complementary feeding is the third most effective action, with the potential to prevent 6 % of deaths in children under 5 years, behind only exclusive breast-feeding and immunisation^(4)^.

Furthermore, early childhood is an extremely important period for the formation of food preferences once the dietary patterns (DP) is established, with the discovery of a variety of textures and flavours, and can be maintained in the subsequent stages of life^(5,6)^. Although DP reflects food preferences, it is influenced by the family context in which the child is inserted, mainly by socio-economic factors^(7,8)^. According to the most recent Brazilian Household Budget Survey (POF)^(9)^, families with lower economic income spend six times less on food expenditure than families with higher income.

Given the huge income inequalities observed in Brazil, associated with the difficult access to basic human development services (education, nutrition and health), from 2003 onwards, the Brazilian government merged several social programmes into a unified conditional cash transfer programme, the Bolsa Família Program (BFP)^(10)^.

In a study that assessed the impact of BFP implementation during its first 5 years, it was identified that the programme contributed to the reduction in the infant mortality rate, particularly owing to the reduction in conditions associated with poverty-related deaths, such as malnutrition and diarrhoea. The authors also reported that the beneficial results of the BFP were related to the conditional adherence of families, particularly children, to health services^(11)^.

However, previous studies point to the higher consumption of unhealthy foods by children assisted by the BFP^(12,13)^. The consumption of ultra-processed foods (UPF) among the most vulnerable populations is possibly related to its low cost and the increase in purchasing power provided by the increase in income by the BFP^(13–15)^.

The first 2 years of life is the stage in which the human being has the highest growth and development rate; therefore, inadequate nutrition during this period can lead to irreversible linear growth impairment, delay in cognitive and motor development, increased risk of overweight, and chronic non-communicable diseases^(1,16–18)^. Hence, this study aimed to identify the DP of children aged 6–24 months assisted by the BFP in Alagoas and verify the association between socio-economic factors and DP.

## Methods

### Design

This is a cross-sectional study and is part of a larger project titled *Evaluation of the Management and Operation of the National Program for Iron and Vitamin A Supplementation and Their Relationship with the Nutritional Status of Children Aged 6–24 Months in Municipalities of Alagoas State*.

### Setting and sample selection

Children aged 6–24 months assisted by the BFP and residents of six municipalities in the State of Alagoas in the north-east region of Brazil were eligible for enrolment. The six municipalities cover all physiographic regions of the State of Alagoas: Pilar (Metropolitan Region), Murici (Zona da Mata), Teotônio Vilela (South Region), São Luís do Quitunde (Northern Region), Pão de Açúcar (Sertão), and Batalha (Agreste) and are included in the present study since their participation in the ‘Early Childhood Program of the State of Alagoas’. This programme has selection criteria to enrol only municipalities that have total coverage of the Brazilian primary care, Family Health Strategy, and at least one team from the Family Health Support Centers (NASF). The state’s early childhood programme aims to contribute to improving the quality of life of pregnant women, nursing mothers and children through intersectoral actions developed in the areas of health, nutrition, education and social assistance. The main socio-demographic characteristics of all municipalities are reported in the online supplementary material.

The calculated sample size was based on the following criteria: expected relative risk for 1:1 ratio of children aged 6–24 months with anaemia and an inadequate minimum dietary diversity according to the study by Saaka and Galaa^(19)^, with 73·2 % of unexposed, 95 % CI and 80 % power. The ratio between the number of children assisted by BFP per municipality and the total number of children assisted by BFP in all six municipalities was calculated to define the sample size in each municipality of the study; therefore, each ratio was individually multiplied by the total sample size. Therefore, the total sample size was calculated to be 1132 children in all 6 municipalities. The number of children assisted by BFP was obtained from the Single Registry system database provided by the State of Alagoas Secretariat of Social Assistance and Development. The sample size was calculated using StatCalc Epi Info version 7.2.2.2 (Center for Diseases Control and Prevention, Atlanta, USA).

Eligible children were identified using the health monitoring map of the BFP-assisted children by municipality, which invited their caregivers to attend the Community Health Centre (CHC) for participation in the study. All families of eligible children were invited by community health workers and through wide dissemination in local media, such as community radio stations or during appointments with health professionals at the CHC. Despite these efforts, a non-probabilistic convenience sampling approach was used, as only children who attended the CHC scheduled for data collection were included. Studies in the municipalities had the consent and partnership of the Municipal and State Health Departments.

Children whose caregivers reported a diagnosis of genetic or autoimmune haematological diseases (sickle cell disease, haemolytic, sideroblastic, and aplastic anaemia, Fanconi anaemia, and thalassemia) or had previously been diagnosed with non-iron deficiency anaemia and nutritionally related type of anaemia (megaloblastic and pernicious anaemia), and those whose mothers had mental health conditions were excluded from the study (*n* 349). In addition, in families with more than one child within the inclusion criteria, only the oldest was selected to participate. In the cases of twin siblings, the included children were randomly selected.

### Data collection

Data were collected between May and December 2018 by trained staff composed of four nutritionists and ten technical assistants. Children’s caregivers were interviewed using a structured questionnaire about socio-demographic, health and environmental issues. Information on family income, sanitation, housing location and the number of residents per household was obtained from secondary data through the Consultation, Selection, and Information Extraction database of the Unified Registry for Social Programs of the Brazilian government (Cadastro Único) using the social registration number (NIS) of each individual provided by the state government.

### Food consumption assessment

The questionnaire for the assessment of food consumption was based on a questionnaire to assess eating habits of children under 2 years of age, as proposed by Oliveira *et al*.^(20)^, which allowed us to verify the consumption of eighteen food and food groups, namely, breast milk, milk (non-breast milk), fruit juice, industrialised (sweetened) juices/soda, coffee/tea, fruits, greens, vegetables, rice/potato/yam/manioc/pasta, beans, meats, eggs, processed meats, cookies/biscuit, instant noodles, porridge, added sugar and candies. The consumption of these food and food groups (yes/no) was referred to the day before the interview. Subsequently, the food groups were classified according to the NOVA^(21)^ classification: fresh/minimally processed foods, processed foods and UPF.

### Household food insecurity

Food insecurity (FI) was assessed using the Brazilian Household Food Insecurity Measurement Scale (EBIA), with fourteen questions (yes or no) about the food-access situation experienced at home in the last 90 d before the interview. Its analysis is based on the sum of the affirmative answers, classified as Household Food Security, and mild, moderate or severe household FI^(22)^.

### Statistical analysis

Data were tabulated by double independent typing and were analysed using the statistical package Stata se^®^ 13.0 (StataCorp.) with a significance level set at 5 %. Socio-economic, demographic, household food security and food consumption variables are described in absolute and relative frequencies. The chi-square test was used to compare socio-economic and consumption of food and food group data according to the municipalities in the study. Bonferroni correction was performed to identify the inter-municipal differences.

Barlett’s test was applied, and the results were statistically significant (*P* < 0·0001). Therefore, the correlation matrix was not an identity matrix, and our food data were suitable for principal component analysis (PCA). All eighteen food group variables were considered in the PCA, and they were configurated with a ‘yes/no’ answer (dummy variable). We used Kaiser–Meyer–Olkin (KMO) to analyse the compliance of variables with PCA.

The number of DP was defined based on eigenvalues higher than 1·1 and eigenvectors (loadings) higher than 0·2. After DP extraction, varimax orthogonal rotation was applied to improve DP interpretation. Values greater than 0·7 are considered acceptable. After setting the DP, a pattern score was calculated for each individual in each pattern. The values of each DP score represented the closeness of the child to DP, and if the DP score was high and positive, the probability of adherence was higher. Each identified DP was considered an outcome variable.

The association between socio-economic and demographic characteristics and standardised DP scores was verified by multilevel linear regression analysis, with random data for each DP outcome. At the individual level, the caregivers’ age and education (years), children’s age and gender, housing location, household per capita income, and household food security were included. All six cities were included at the contextual level. The theoretical model proposed to assess factors associated with children’s DP is reported in the online supplementary material (see online Supplemental Fig. S1). The intraclass correlation coefficient was estimated to calculate the proportion of variance explained by the different characteristics between cities.

## Results

This study included 1604 children aged 6–24 months. Table 1 shows the socio-demographic characteristics of the municipalities. The percentage of the number of residents assisted by the BFP ranged from 59·3 % in Batalha to 40·1 % in Teotônio Vilela. All municipalities had a low level of Human Development Index (HDI), except for Pilar (average HDI), and urbanisation rate above 60·0 %, except for Pão de Açúcar (45·2 %).

**Table 1:** The main socio-demographic characteristics of all municipalities participating in the study, Alagoas, Brazil, 2018

	Batalha	Murici	Pão de Açúcar	Pilar	São Luís do Quitunde	Teotônio Vilela
	%	%	%	%	%	%
Population*						
* n*	17 079	26 710	23 811	33 305	32 412	41 152
Population density*	53·21	62·58	34·86	133·37	81·61	138·15
Human Development Index*	0·594	0·527	0·593	0·610	0·536	0·564
Urbanisation rate*	70·5	82·5	45·2	95·5	63·5	84·5
Number of people assisted by BFP						
* n*	10 122	12 712	10 980	15 067	16 786	16 502
* *%†	59·3	47·6	46·1	45·2	51·8	40·1
Number of children (< 2 years) assisted by BFP						
* n*	370	554	420	527	612	554
* *%†	2·2	2·1	1·8	1·6	1·9	1·3

BFP, Bolsa Família Program.

* Data obtained from the Brazilian Demographic Census 2010 (IBGE, 2011).

† Data obtained from the Consultation, Selection, and Information Extraction database (CECAD) provided by the state government of Alagoas.

Regarding the socio-economic and demographic characteristics of the children (Table 2), 85·0 % lived in urban areas, approximately 93·0 % of the families were in extreme poverty, 41·5 % of the caregivers had less than 9 years of study and 66·5 % of the families were in FI. The prevalence of FI in São Luís do Quitunde (76·6 %) was significantly higher than that in Murici (58·0 %), Pilar (62·6 %) and Teotônio Vilela (65·0 %). The prevalence of FI in Batalha and Pão de Açúcar did not differ from the others.

**Table 2:** Socio-economic and demographic characteristics of children aged 6–24 months assisted by the Bolsa Família Program and their families in the State of Alagoas, Brazil, 2018

	Total		Batalha		Murici		Pão de Açúcar		Pilar		São Luís do Quitunde		Teotônio Vilela		*χ*^2^ test
	*n*	%	*n*	%	*n*	%	*n*	%	*n*	%	*n*	%	*n*	%	*P*-value
Household characteristics															
Household per capita income*,†															
USD ≤ 21·90	1490	92·9	192^a^	97·0	284^a^	94·7	184^a^	96·8	289^a,b^	93·2	294^a,b^	91·9	247^b^	86·4	<0·001
USD 21·91–43·81	88	5·5	4^a^	2·0	9^a^	3·0	4^a^	2·1	18^ab^	5·8	17^a^	5·3	36^b^	12·6	
USD 43·82–122·94‡	26	1·6	2^a^	1·0	7^a^	2·3	2^a^	1·1	3^a^	1·0	9^a^	2·8	3^a^	1·0	
Housing location*															
Urban	1364	85	104^a^	52·5	274^b^	91·3	111^a^	58·4	302^c^	97·4	293^b^	91·6	280^c^	97·9	<0·001
Rural	240	15	94^a^	47·5	26^b^	8·7	79^a^	41·6	8^c^	2·6	27^b^	8·4	6^c^	2·1	
Household food security															
Food security	537	33·5	35^a,b,c,d,e^	32·8	126^d,e^	42·0	55^a,b,c,d,e^	28·9	116^c,e^	37·4	75^b^	23·4	100^a,c,d,e^	35·0	<0·001
Food insecurity	1067	66·5	133^a,b,c,d,e^	67·2	174^d,e^	58·0	135^a,b,c,d,e^	71·1	194^c,e^	62·6	245^b^	76·6	186^a,c,d,e^	65·0	
Caregiver characteristics															
Age (years)															
< 19	346	21·6	29^a^	14·6	64^a,b^	21·3	30^a^	15·8	67^a,b^	21·6	98^b^	30·6	58^a,b^	20·5	<0·001
19–35	1083	67·6	147^a^	74·2	201^a,b^	67·0	148^a^	77·9	204^a,b^	65·8	192^b^	60·0	191^a,b^	67·5	
> 35	172	10·7	22^a^	11·1	35^a^	11·7	12^a^	6·3	39^a^	12·6	30^a^	9·4	34^a^	12·0	
Education (years)															
No education	94	6·0	10^a,b,c,d,e^	5·1	24^d,e^	8·5	17^c,e^	8·9	6^b^	1·9	13^a,b,c,d,e^	4·1	24^a,c,d,e^	8·4	<0·001
< 9	553	35·5	60^a^	30·8	116^a^	41·3	34^b^	17·9	111^a^	35·8	122^a^	38·2	110^a^	38·6	
9 or 12	861	55·3	112^a,b,c^	57·4	139^c^	49·5	125^b^	66·3	163^a,b,c^	52·6	177^a,b,c^	55·5	144^a,c^	50·5	
> 12	48	3·1	13^a^	6·7	2^b^	0·7	13^a^	6·8	6^a,b^	1·9	7^a,b^	2·2	7^a,b^	2·5	
Child characteristics															
Gender															
Female	806	50·2	97	49·0	141	47·0	97	51·1	163	52·6	169	52·8	139	48·6	0·659
Male	798	49·8	101	51·0	159	53·0	93	48·9	147	47·4	151	47·2	147	51·4	
Age (months)															
6–11	520	32·4	79	39·9	106	35·3	53	27·9	97	31·3	99	30·9	56	30·1	0·189
12–17	554	34·5	54	27·3	103	34·3	74	38·9	117	37·7	106	33·1	100	35·0	
18–24	530	33·0	65	32·8	91	30·3	63	33·2	96	31·0	115	35·9	100	35·0	

* Data obtained from the Consultation, Selection, and Information Extraction database (CECAD) provided by the state government of Alagoas.

† Household per capita income does not include the value received by the Bolsa Família Program.

‡ Amount referring to half the minimum wage inreais in 2018 (minimum wage in 2018 = R$ 954·00); in December 2018, $1·00 USD was approximately R$ 3·88.

^a,b,c,d,e^Bonferroni correction: Values with different superscripts are significantly different, with an adjusted *P*-value < 0·003.

Regarding food intake (Table 3), 59·0 % of the children were still breast-feeding, and 80·6 % consumed beans, 75·7 %, fruits, 68·1 %, meats, and only 21·9 %, eggs. The municipality of Pão de Açúcar had a significantly higher prevalence of consumption of beans (100 %) and eggs (46·8 %) compared to other municipalities. Notably, 57·6 % of the children consumed the added sugar.

**Table 3:** Distribution of dietary practices of children aged 6–24 months assisted by the Bolsa Família Program in the State of Alagoas, Brazil, 2018

	Total (*n* 1·604)		Batalha		Murici		Pão de Açúcar		Pilar		SãoLuís do Quitunde		Teotônio Vilela		*χ*^2^ test
	*n* *	%*	*n* *	%*	*n* *	%*	*n* *	%*	*n* *	%*	*n* *	%*	*n* *	%*	*P*-value
Variable															
Breast milk	947	59·0	128^a,b,c^	64·6	181^c,d^	60·3	144^b^	75·8	155^d^	50·0	160^d^	50·0	179^a,c^	62·6	<0·001
Milk (non-breast milk)	1097	68·4	146^a^	73·7	176^b^	58·7	133^a,b^	70·0	216^a,b^	69·7	229^a^	71·6	197^a,b^	68·9	0·003
Fruit juices	958	59·7	109^a^	55·1	182^a,b^	60·7	134^b^	7·5	179^a,b^	57·7	181^a^	56·6	173^a,b^	60·5	0·023
Industrialised (sweetened) juices/soda	248	15·5	13^a^	6·6	48^b^	16·0	41^b^	21·6	52^b^	16·8	44^a,b^	13·8	50^b^	17·5	0·002
Coffee/tea	404	25·2	49^a,b,c^	24·7	76^c^	25·3	104^d^	54·7	45^b,e^	14·5	39^e^	12·2	91^a,c^	31·8	<0·001
Fruits	1215	75·7	134^a^	67·7	222^a,b^	74·0	170^c^	89·5	235^a,b^	75·8	218^a^	68·1	236^b,c^	82·5	<0·001
Greens	269	16·8	41^a,b^	20·7	38^b,c^	12·7	33^a,b^	17·4	74^a^	23·9	25^c^	7·8	58^a,b^	20·3	<0·001
Vegetables	926	57·7	112	56·5	183	61·0	109	57·4	169	54·5	172	53·8	181	63·3	0·133
Rice, potato, yam, manioc or pasta	1322	82·4	161	81·3	255	85·0	155	81·6	256	82·6	248	77·5	247	86·4	0·074
Beans	1293	80·6	171^a^	86·4	239^a,b,c^	79·7	190^d^	100·0	234^b,c^	75·5	226^c^	70·6	233^a,b^	81·5	<0·001
Meats	1092	68·1	138^a,b,c^	69·7	193^a,b,c^	64·3	144^c^	75·8	210^a,b,c^	67·7	196^b^	61·3	211^a,c^	73·8	0·002
Eggs	352	21·9	34^a^	17·2	61^a^	20·3	89^b^	46·8	55^a^	17·7	56^a^	17·5	57^a^	19·9	<0·001
Processed meats	203	12·7	12^a^	6·1	45^b,c^	15·0	44^c^	23·2	29^a,b^	9·4	43^a,b,c^	13·4	30^a,b^	10·5	<0·001
Cookies/biscuits	1200	74·8	155	78·3	211	70·3	153	80·5	237	76·5	238	74·4	206	72·0	0·094
Instant noodles	135	8·4	8^a^	4·0	15^a^	5·0	36^b^	18·9	28^a^	9·0	32^a,b^	10·0	16^a^	5·6	<0·001
Porridge	381	23·8	52^a^	26·3	56^a,b^	18·7	87^c^	45·8	39^b^	12·6	87^a^	27·2	60^a,b^	21·0	<0·001
Added sugar	924	57·6	107^a,b,c^	54·0	184^c,d^	61·3	132^d^	69·5	149^b^	48·1	197^a,c,d^	61·6	155^a,b,c^	54·2	<0·001
Candies	364	22·7	37^a,b^	18·7	56^a,b^	18·7	79^c^	41·6	40^b^	12·9	88^a^	27·5	64^a^	22·4	<0·001

* Values are presented as the total number (*n*) and frequency (%) for categorical variables.

^a,b,c,d,e^Bonferroni correction: Values with different superscripts are significantly different, with an adjusted *P*-value < 0·003.

Four DP were identified (Table 4). The first dietary pattern (DP1) was composed mainly of staple foods of the traditional Brazilian diet (vegetables, rice, potatoes, yams, manioc, pasta, beans, meats and cookies/biscuits). The second dietary pattern (DP2) was formed mainly by industrialised juice/soft drinks, processed meats, instant noodles and candies, except for eggs and coffee/tea. The third dietary pattern (DP3) consisted of replacing breast milk with other types of milk (non-breast milk) with added sugar. Finally, the fourth dietary pattern (DP4) comprised only fresh and minimally processed foods (fruit juice, fruits and green).

**Table 4:** Factor loading matrix of the extracted dietary patterns for children aged 6–24 months assisted by the Bolsa Família Program in the State of Alagoas, Brazil, 2018

Variable	DP1*	DP2†	DP3‡	DP4§
Breast milk			−0·6078	
Milk (non-breast milk)			0·6460	
Fruit juices				0·6432
Industrialised (sweetened) juices/soda		0·3716		−0·3186
Coffee/tea		0·3968		
Fruits				0·5277
Greens				0·2399
Vegetables	0·3401			0·2387
Rice, potato, yam, manioc or pasta	0·4946			
Beans	0·4986			
Meats	0·4736			
Eggs		0·3024		0·2461
Processed meats		0·4324		
Cookies/biscuits	0·2694			
Instant noodles		0·3655		
Porridge				
Added sugar		0·2058	0·3932	
Candies		0·4276		
Eigenvalues	2·26	1·94	1·66	1·44
Variance				
%	12·5	10·8	9·2	8·0
KMO	0·7321			

DP, dietary patterns; KMO, Kaiser–Meyer–Olkin.

* DP1: Traditional Brazilian food.

† DP2: Ultra-processed foods.

‡ DP3: Milk (non-breast milk) with added sugar.

§ DP4: Fresh and minimally processed foods.

Table 5 shows the association between the socio-economic and demographic characteristics of the children and DP. Household per capita income was negatively associated with DP1 (*β* = −0·794; (95 % CI −1·351, −0·236)). Younger (*β* = −0·008; (95 % CI −0·017, −0·000)) and less educated caregivers (*β* = −0·037; (95 % CI −0·056, - 0·018)) were associated with DP2. A negative association was found between household per capita income and DP3 (*β* = −0·502; (95 % CI −0·995, 0·009)). We also observed a negative association between the families’ FI situation (*β* = −0·204; (95 % CI −0·331, −0·078)) and DP4 and a positive association between age (*β* = 0·011; (95 % CI 0·003, 0·019)) and caregivers’ education (*β* = 0·043; (95 % CI 0·025, 0·061)). Multilevel analysis revealed that 12 % of the variation in the DP2 score was explained by the municipality where the child and the family lived (Table 5). DP2 was the DP with the greatest variation explained by the municipality of residence.

**Table 5:** Association by multilevel linear regression analysis between socio-economic and demographic characteristics and dietary patterns of children aged 6–24 months assisted by the Bolsa Família Program and their families in the State of Alagoas, Brazil, 2018

Variable	DP1			DP2			DP3			DP4		
	*β*	95 % CI	*P*-value	*β*	95 % CI	*P*-value	*β*	95 % CI	*P*-value	*β*	95 % CI	*P*-value
Caregiver age (years)	0·001	−0·008, 0·011	0·792	−0·008	−0·017, −0·000	0·046	−0·003	−0·011, 0·005	0·448	0·011	0·003, 0·019	0·003
Caregiver education (years)	0·012	−0·009, 0·034	0·275	−0·037	−0·056, −0·018	<0·001	0·004	−0·014, 0·024	0·622	0·043	0·025, 0·061	<0·001
Housing location*,†	0·102	−0·118, 0·322	0·363	0·189	−0·008, 0·386	0·060	0·056	−0·133, 0·247	0·558	0·025	−0·154, 0·206	0·779
Household per capita income*,‡												
USD ≤ 21·90	Ref.			Ref.			Ref.			Ref.		
USD 21·91–43·81	0·000	−0·313, 0·314	0·996	0·147	−0·129, 0·423	0·297	0·084	−0·193, 0·361	0·552	0·130	−0·126, 0·386	0·320
USD 43·82–122·94§	−0·794	−1·351, −0·236	0·005	−0·044	−0·533, 0·445	0·860	−0·502	−0·995, −0·009	0·046	−0·118	−0·572, 0·335	0·609
Household food security||	0·069	−0·085, 0·224	0·381	0·061	−0·075, 0·197	0·379	0·114	−0·022, 0·251	0·102	−0·204	−0·331, −0·078	0·002
Child gender¶	−0·073	−0·215, 0·069	0·315	−0·136	−0·262, −0·011	0·032	0·088	−0·037, 0·214	0·168	−0·056	−0·172, 0·059	0·342
Child age (months)	0·080	0·066, 0·094	<0·001	0·080	0·067, 0·092	<0·001	0·032	0·020, 0·045	<0·001	0·020	0·008, 0·031	0·001
ICC	0·021			0·120			0·008			0·029		
95 % CI	0·005, 0·076			0·040, 0·306			0·001, 0·041			0·007, 0·100		

DP, dietary patterns; Ref, reference variable; ICC, intraclass correlation coefficient.

* Data obtained from the Consultation, Selection, and Information Extraction database (CECAD) provided by the state government of Alagoas.

† Dummy variable (urban and rural). The urban variables were defined as the reference category.

‡ Household per capita income does not include the value received by the Bolsa Família Program.

§ Amount referring to half the minimum wage inreais in the year of 2018 (minimum wage in 2018 = R$ 954·00); in December 2018, $1·00 USD was approximately R$ 3·88.

|| Dummy variable (food security and insecurity). The food security variable is defined as the reference category.

¶ Dummy variable (females and males). The female variable was defined as the reference category.

## Discussion

We highlight the two main results of our study. DP2, mainly compounded by UPF, was associated with the caregivers’ minor age and lower education, while DP4, compounded by a healthier diet based on fresh and minimally processed foods, was associated with household food security and with caregivers with higher education and older age.

These findings point to the presence of socio-economic inequities related to the DP of children under 2 years of age assisted by BFP in the State of Alagoas. This indicates that, on its own, the BFP is not sufficient to guarantee the food and nutritional security of individuals. Apparently, only quantitative access to food is achieved, while qualitative access is impaired by the set of socio-economic factors in which individuals are inserted.

Similar to our study, other authors have also associated DP characterised by the presence of UPF (DP2) with low socio-economic status, lower educational level and maternal age^(23–26)^. This indicates that the caregivers’ higher education is related to greater purchasing power and access to health information, which can lead to healthier food choices and purchases. In addition, considering the massive advertising of UPF by the food industry, caregivers with older age and higher educational level may have a greater capacity to critisise and lower vulnerability to media influences^(27–29)^. These data support the association found between the healthiest DP, composed of fresh and minimally processed foods (DP4), with the best socio-demographic indicators. We also highlight that DP4 presented the least variance explanation in the PCA. The consumption of fresh and minimally processed foods may indicate greater dedication to the preparation of the food itself. Martins *et al.*^(30)^ showed that children of parents with culinary skills consume less UPF, which reinforces the role of cooking as a tool for the protection and promotion of adequate and healthy food consumption. According to the Dietary Guidelines for Brazilian children under 2 years old^(31)^, an adequate and healthy diet should be based on the consumption priority of fresh or minimally processed foods, while the UPF should not be offered to children under 2 years old.

Another pattern observed in this study is that DP1, characterised by both the staple foods of the traditional Brazilian diet and higher variance percentage explained in the PCA, can also be classified as healthy. However, Souza *et al.*^(32)^ emphasise that this can be considered a monotonous pattern because although it is composed of necessary foods for adequate infant nutrition, it does not include fruits and greens, which in our study belonged to DP4. In contrast, DP1 also encompasses a group of vegetables, an ingredient often used in local culture for the preparation of porridge.

Although DP1 is usually reported as traditional by studies focusing on the PCA for assessing food consumption, it presents considerable differences in food groups concerning the local culture where the studies are conducted. In addition, this type of DP is commonly related to low-income families^(33–38)^. Moreover, in the present study, DP1 was inversely associated with the highest per capita income, indicating maintenance of the regional food culture, mainly composed of the consumption of roots (manioc, sweet potatoes and yams), vegetables, and the typical Brazilian combination of ‘beans and rice.’ Although this aspect was not evaluated, considering the local culture, it is possible to infer that the maintenance of this regional food culture is related to family farming, whose families have the habit of planting roots. Also noteworthy is the usual offer of cookies/biscuits (UPF), reaching an alarming consumption frequency of almost 75 % among the children studied.

An increase in the consumption of UPF has been observed in recent decades in Brazil and worldwide. The new food system, shaped by international policies to promote the flow of capital and the rapid expansion of trade, opened the market for the establishment of large food industries and fast-food chains^(39,40)^. In addition, marketing and the massive influence of the media have contributed to changes in the design of an ideal diet^(41)^. The changes in the current food system are reflected in culinary practices with the search for greater practicality in preparing meals. Thus, previous studies indicate that the traditional Brazilian diet, based on the consumption of fresh food, minimally processed foods, and processed culinary ingredients, has been gradually replaced by UPF^(42–45)^.

Studies conducted with children assisted by BFP observed that despite the programme having enhanced food diversity, it also leads to unhealthy food choices, with greater consumption of UFP^(12,13,46)^. Marçal *et al.*^(46)^ observed a high consumption of UPF with discontinuation of breast-feeding among children under 2 years of age who were assisted by BFP. Moreover, as much as the increase in income provided by the BFP’s income transfer leads beneficiaries to greater access to food, it does not guarantee its adequacy in terms of nutritional quality. Fresh and minimally processed foods, such as meats, fruits, greens and vegetables, in general, have the highest cost, which means that families tend to buy more UPF, which is usually hyper-palatable and has a lower cost^(13,47)^. However, we highlight the importance and necessity of health promotion and healthy nutrition actions conducted by the CHC workers during the monitoring of compliance with the BFP conditionalities in the health field, such as monitoring child growth and development and vaccination of children under 7 years old.

The consumption of UPF in the window of the first 1000 d, a period that includes the child’s conception until the second year of life, may result in excessive body weight gain and thus damage child growth (stunting), resulting in the phenomenon known as the double burden of malnutrition^(41,48–51)^. Double burden of malnutrition is characterised by the coexistence of undernutrition (micronutrient deficiency, low weight for age, stunting and wasting), overweight and non-communicable diseases related to food^(41,48)^. Exposure to malnutrition in all its forms during this critical period of child development can result in loss of human potential, with the perpetuation of the intergenerational cycle of poverty and malnutrition^(3,48)^.

Finally, the association of DP3, which consisted of replacing breast milk with other types of milk (non-breast milk) with added sugar, was inversely associated with a higher per capita household income range. In contrast to this observation, it has been reported that breast-feeding should be even more prioritised when financial resources are insufficient for the acquisition of food, avoiding unnecessary expenses with the acquisition of other sources of milk^(52,53)^. However, Bayoumi *et al.*^(54)^ observed that children from low-income families are more likely to be fed infant formula in the first year of life and drink more than two cups of cow’s milk/d, along with a shorter duration of breast-feeding. Conversely, Victora *et al.*^(55)^ observed that in 98 low- and middle-income countries, mothers with lower economic income were more likely to breastfeed longer than mothers with higher income. Following the same rationale, Lamounier *et al.*^(56)^ associated the shorter duration of breast-feeding with higher levels of maternal education and higher income quintiles in five Latin American countries. However, despite the differences found between the per capita income brackets, it is noteworthy that our entire sample lived in extreme poverty, which can explain, at least in part, the differences observed.

Many studies have shown a high frequency of consumption of other types of milk at an early age, with cow’s milk as the most consumed type^(31,57,58)^. According to the Dietary Guidelines for Brazilian children under 2 years old^(31)^, on average, two-thirds of Brazilian children under 6 months of age have already received another type of milk, mainly cow’s milk, which is usually supplemented with sugar and flour. Wijndaele *et al*.^(58)^ associated the early introduction of cow’s milk to a family with low socio-economic level. Therefore, the importance of strengthening the actions promoted by the National Strategy for the Promotion of Breastfeeding and Healthy Complementary Food in the Brazilian Unified Health System (SUS) – the Brazilian Breastfeeding and Complementary Feeding Strategy – is emphasised to qualify the work process of primary care professionals that reinforces and encourages the promotion of breast-feeding and healthy eating habits for children under 2 years of age within the scope of the SUS^(59)^.

It is important to consider that the present study has some limitations. First, the questionnaire method used to assess infant feeding practices has potential memory response bias and may represent an atypical feeding day that does not represent long-term eating patterns. However, we followed the recommended research method using a questionnaire for the evaluation of the WHO indicators for assessing infant and young child feeding practices^(60)^. This method is widely used and is considered appropriate in food consumption studies when the objective is to describe infant feeding practices in populations^(22)^. Another limitation is the non-probabilistic sampling approach, which may cause selection bias, including only those who have more access to health services and possibly greater health care. However, we reinforce that all eligible children were invited to participate in the study, and the data collection was performed in all CHC in the municipalities, both in urban and rural areas. However, because of the diversity of DP, it is assumed that the results would not be different.

Despite the limitations observed, this study provides important information regarding the DP of children under 2 years of age assisted by BFP in the State of Alagoas. We highlight the identification of DP through PCA in an age group in which studies with this approach are scarce. Given the socio-economic similarities of the studied population with those of other parts of the country and the world, the results of this study could be extrapolated to other regions.

In conclusion, this study shows the influence of socio-demographic characteristics, mainly FI and caregivers’ low age and education, on the formation of eating patterns early in life in children assisted by BFP in the State of Alagoas. Given that food choices are not only guided by economic conditions but also multi-determined and conditioned by different aspects, such as the influence of media, climatic conditions, psychosocial, educational, cultural, political, ethical and religious factors^(13,61–63)^, the present findings reinforce the need to strengthen and/or implement intersectoral public policies together with the BFP. Attending this matter is highly necessary to guarantee the Human Right to Adequate Food at promoting food and nutritional security and actions that include the regulation of unhealthy food dissemination, thus contributing to health promotion and combating double burden of malnutrition.

## Supplementary material

For supplementary material accompanying this paper visit https://doi.org/10.1017/S1368980021004110. click here to view supplementary material
