# Supplementary material for: Dietary patterns of children aged 6–24 months assisted by the Bolsa Família Program
Source: Public Health Nutr. 2021 Oct 1;25(10):2794–804. doi: 10.1017/S1368980021004110 (PMC9991842; doi:10.1017/S1368980021004110)
Supplement: Supplementary file 1 [file S1368980021004110sup001.docx]

**Supplemental Figure S1.** Theoretical model proposed to assess factors associated with dietary patterns of children aged 6–24 months, assisted by the Bolsa Família Program and their families in the State of Alagoas, Brazil, 2018.

**LEVEL 2**

Municipalities participating in the study

DP1

DP2

DP3

DP4

**LEVEL 1**

**Household characteristics**

Household per capita income Housing location Household food security

**Caregiver characteristics**

Age (years) Education (years)

**Child characteristics**

Gender Age (months)
